# Supplementary material for: Utilization Trend and Comparison of Different Radiotherapy Modes for Patients with Early-Stage High-Intermediate-Risk Endometrial Cancer: A Real-World, Multi-Institutional Study
Source: Cancers (Basel). 2022 Oct 19;14(20):5129. doi: 10.3390/cancers14205129 (PMC9599971; doi:10.3390/cancers14205129)
Supplement: Supplementary file 1 [file cancers-14-05129-s001.zip › cancers-1947714-supplementary.pdf]

Supplementary Materials: Utilization Trend and Comparison of Different Radiotherapy Modes for Patients with Early-Stage High–Intermediate-Risk Endometrial Cancer: A Real-World, Multi-Institutional Study

Kang Ren, Lijuan Zou, Tiejun Wang, Zi Liu, Jianli He, Xiaoge Sun, Wei Zhong, Fengju Zhao, Xiaomei Li, Sha Li, Hong Zhu, Zhanshu Ma, Shuai Sun, Wenhui Wang, Ke Hu, Xiaorong Hou, Lichun Wei and Fuquan Zhang

Table S1. Subgroup analyses in GOG249 HIR group

			**Before matching**				**After matching**			
			**OS**	**DFS**	**DMFS**	**LRFS**	**OS**	**DFS**	**DMFS**	**LRFS**
**GOG-249**	**cN0**	**VBT**	85.40%	62.30%	71.50%	72.80%	72.80%	62.30%	71.50%	72.80%
		**EBRT±VBT**	91.60%	87.40%	87.40%	92.10%	96.20%	93.00%	93.00%	96.20%
		P	0.236	**0.012**	**0.036**	**0.014**	0.194	**0.049**	0.084	**0.044**
	**pN0**	**VBT**	95.70%	91.60%	93.40%	94.50%	95.70%	93.50%	93.40%	94.50%
		**EBRT±VBT**	91.20%	87.40%	87.80%	92.00%	90.10%	81.60%	82.60%	89.40%
		P	0.626	0.548	0.329	0.92	0.617	0.192	0.108	0.869
**Exclude FIGO II from GOG-249 cohort**	**cN0**	**VBT**	85.40%	62.30%	71.50%	72.80%	85.40%	62.30%	71.50%	72.80%
		**EBRT±VBT**	89.50%	88.10%	88.10%	90.10%	86.10%	86.30%	86.20%	90.90%
		P	0.431	**0.025**	0.057	0.057	0.847	0.103	0.174	0.128
	**pN0**	**VBT**	95.60%	93.40%	95.20%	94.40%	95.60%	93.40%	95.20%	94.30%
		**EBRT±VBT**	90.90%	90.60%	90.50%	93.00%	89.50%	90.70%	90.60%	92.10%
		P	0.665	0.896	0.55	0.779	0.678	0.928	0.681	0.714

Table S2. Acute toxicities for patients treated with EBRT±VBT and V

	Whole patients(N=1038)			GOG-249 HIR(N=473)			PORTEC-2 HIR(N=184)			ESMO-ESGO-ESTRO HIR(N=207)		
	**EBRT±VBT**	**VBT**	***P***	**EBRT±VBT**	**VBT**	***P***	**EBRT±VBT**	**VBT**	**P**	**EBRT±VBT**	**VBT**	**P**
**Hematological**												
0	317(55.1%)	435(94.0%)	<0.001	178（54.8%）	136（91.9%）	<0.001	61(68.5%)	89(93.7%)	<0.001	55（57.3%）	94（90.4%）	<0.001
1–2	231 (40.2%)	28(6.0%)		134（41.2%）	12（8.1%）		28(31.5%)	6(6.3%)		38（39.6%）	10（9.6%）	
3–4	27(4.7%)	0(0)		13（4.0%）	0（0.0%）					3（3.1%）	0（0）	
**Upper GI**												
0	334 (58.1%)	420(90.7%)	<0.001	190a（58.5%）	130（87.8%）	<0.001	190(58.5%)	130(87.8%)	<0.001	53（55.2%）	89（85.6%）	<0.001
1–2	239(41.6%)	43(9.3%)		134（41.2%）	18（12.2%）		134(41.2%)	18(12.2%)		43(44.8%)	15(14.4%)	
3	2(0.3%)	0(0%)		1（0.3%）	0a（0.0%）		1(0.3%)	0(0)		0	0	
**Lower GI**												
0	185a（32.2%）	377（81.4%）	<0.001	115（35.4%）	117b（79.1%）	<0.001	34（38.2%）	81（85.3%）	<0.001	30（31.3%）	75（72.1%）	<0.001
1–2	386（67.1%）	86（18.6%）		206（63.4%）	31（20.9%）		54（60.7%）	14（14.7%）		66（68.8%）	29（27.9%）	
3	4（0.7%）	0a（0.0%）		4（1.2%）	0（0.0%）		1（1.1%）	0（0%)				
**Urinary Tract**												
0	464（51.4%）	438（48.6%）	<0.001	274a（84.3%）	137（92.6%）	0.044	70a（78.7%）	90（94.7%）	0.0047	75（78.1%）	100（96.2%）	<0.001
1–2	110（81.5%）	25（18.5%）		50（15.4%）	11（7.4%）		17（19.1%）	4（4.2%）		21（21.8%）	4（3.8%）	
3	1（100.0%）	0（0.0%）		1（0.3%）	0（0.0%）		2（2.2%）	1（1.1%）				

Note：GI gastrointestinal, EBRT External Beam Radiation, VBT Vaginal Brachytherapy.
